# A case of polymicrobial anaerobic spondylodiscitis due to *Parvimonas micra* and *Fusobacterium nucleatum*

**DOI:** 10.1099/jmmcr.0.005092

**Published:** 2017-04-26

**Authors:** Leanne M. Cleaver, Shara Palanivel, Damien Mack, Simon Warren

**Affiliations:** ^1^​ Royal Free London NHS Foundation Trust, London, UK; ^2^​ Royal National Orthopaedic Hospital, Stanmore, London, UK

**Keywords:** spondylodiscitis, polymicrobial discitis, *Fusobacterium nucleatum*, *Parvimonas micra*, MALDI-TOF

## Abstract

**Introduction.** Here, we present a case of polymicrobial anaerobic spondylodiscitis.

**Case Presentation.** A forty-five year-old female patient was referred to a specialist orthopaedic hospital with an eight week history of back pain without fevers. X-ray imaging and magnetic resonance imaging showed acute osteomyelitis of the twelfth thoracic and first lumbar vertebrae. Prolonged enrichment cultures grew *Parvimonas micra* and *Fusobacterium nucleatum*, identified by matrix-assisted laser desorption ionisation-time of flight (MALDI-ToF) mass spectrometry (MS). The patient was successfully treated with six weeks of intravenous ertapenem and oral clindamycin.

**Conclusion.** Anaerobic discitis is rare, and polymicrobial discitis is rarer still. A PubMed literature review revealed only seven cases of *F. nucleatum* discitis and only twelve cases of *P. micra* discitis; this includes only one other reported case of a polymicrobial discitis due to infection with both anaerobes. We emphasise the importance of prolonging enrichment culture and the use of fast yet accurate identification of anaerobes using MALDI-ToF MS in these infections.

## Abbreviations

CT, computed tomography; MALDI-TOF, matrix-assisted laser desorption ionisation-time of flight; MRI, magnetic resonance imaging.

## Introduction

*P. micra*, a Gram-positive, slow-growing, anaerobic coccus, was once part of the *Peptostreptococcus*, *Peptococcus* and *Micromonas* genus of bacteria before being relatively recently reclassified as *Parvimonas* [1]. It is typically found in the human oral cavity and gastrointestinal tract. *F.* *nucleatum* is a Gram-negative, non-spore forming, slow-growing, anaerobic bacillus. This anaerobe is also typically found in the human oral cavity and the gastrointestinal tract, but is also, controversially, reported to be found in the female genital tract [2].

Both *P. micra* and *F. nucleatum* have been implicated in contiguous infections, causing abscesses in the maxillofacial region and the abdomen. These anaerobes have also been implicated in more invasive infections, including long bone and vertebral osteomyelitis. A study by Grammatico *et al.* in 2008 reported that in France spondylodiscitis cases occur in 2.4 per 100 000 people, which is comparable to other Western countries [1]. Vertebral osteomyelitis of anaerobic aetiology is very rare – approximately 3–4 % of all spondylodiscitis cases are due to anaerobic organisms [2, 3]. Polymicrobial spondylodiscitis cases are rarer still – they account for 1.5 % of discitis cases [4].

The difficulty in diagnosis of anaerobic infections causing vertebral osteomyelitis may be due to the current conventional methods used in most microbiology laboratories, such as Robinson’s cooked meat (RCM) enrichment and short incubation times for cultures, which can impede microbiological diagnosis [5]. Here, we describe a case of successfully treated spondylodiscitis caused by *P. micra* and *F. nucleatum* identified by prolonged incubation using the Bactec (Becton Dickinson, USA) blood culture system and matrix-assisted laser desorption ionisation-time of flight (MALDI-ToF) mass spectrometry (MS) (Bruker, Germany).

## Case report

A forty-five year old Caucasian female was referred to the Royal National Orthopaedic Hospital, Stanmore, UK, due to an eight week history of lumbar back pain after lifting a bag onto an overhead luggage rack. The pain did not respond to analgesia or physiotherapy. During this time, the patient experienced no fevers, sweats or weight loss. The patient recalled no urinary symptoms, had never had any other back pain or injury, no epidural anaesthetic, no skin conditions, no sinusitis or otitis media, no gastrointestinal issues and no other significant co-morbidities, such as diabetes. The patient had undergone surgery as a teen to remove varicose veins in the leg due to Klippel–Trenaunay syndrome; a congenital vascular disorder where one limb may be affected by port wine staining, varicose veins or too much bone and soft tissue growth. She had received no antibiotics in recent memory. Some years previously, the patient had had a dental cavity filling and some subsequent dental hygienist procedures, but she had good oral health. She had a copper intrauterine device *in situ* since 2009.

The patient was born and raised in Australia, and moved to the United Kingdom twenty years previously, where she resided in London working as a consultant in finance. Recent travel history included visiting her father in hospital in 2014 in Manila in the Philippines. The patient was a smoker (fifteen cigarettes a day) and drank approximately fourteen units of alcohol per week. Other than the aforementioned, the patient was healthy and well.

**Fig. 1.**
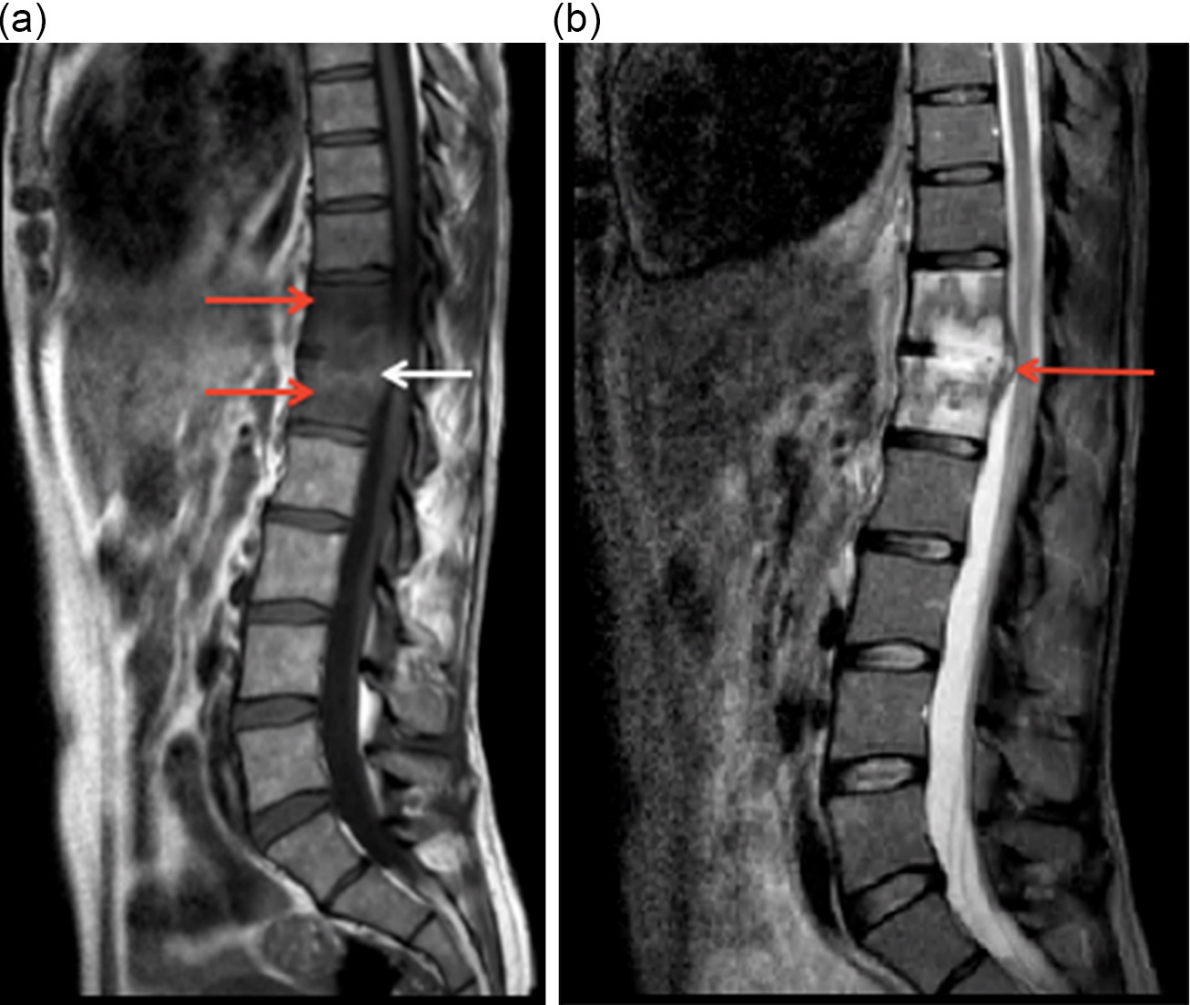
(a) T1-weighted sagittal MRI scan. Red arrows indicate areas of marrow oedema. The white arrow indicates a hyperintense rim (penumbra sign) surrounding the fluid signal intensity within the T12–L1 disc space. (b) STIR (short T1 inversion recover) sagittal MRI scan. The red arrow indicates the extension of disc space fluid.

The patient was referred to our specialist orthopaedic hospital based on abnormal outpatient imaging of the spine. The patient’s C-reactive protein (CRP) at the time of admission was <10 mg l^−1^ and her white cell count was within the normal range. T1-weighted sagittal magnetic resonance imaging (MRI) of the thoracolumbar spine showed reduced signal intensity of the T12 and L1 vertebral bodies that indicated marrow oedema, and destruction of the central and posterior aspect of the T12–L1 intervertebral disc and endplates (Fig. 1a). Short T1 inversion recover (STIR) sagittal imaging also showed a small posterior extension of disc space fluid, elevating the posterior longitudinal ligament (Fig. 1b). These images demonstrated discitis of the twelfth thoracic and first lumbar vertebrae.

## Investigations

**Fig. 2.**
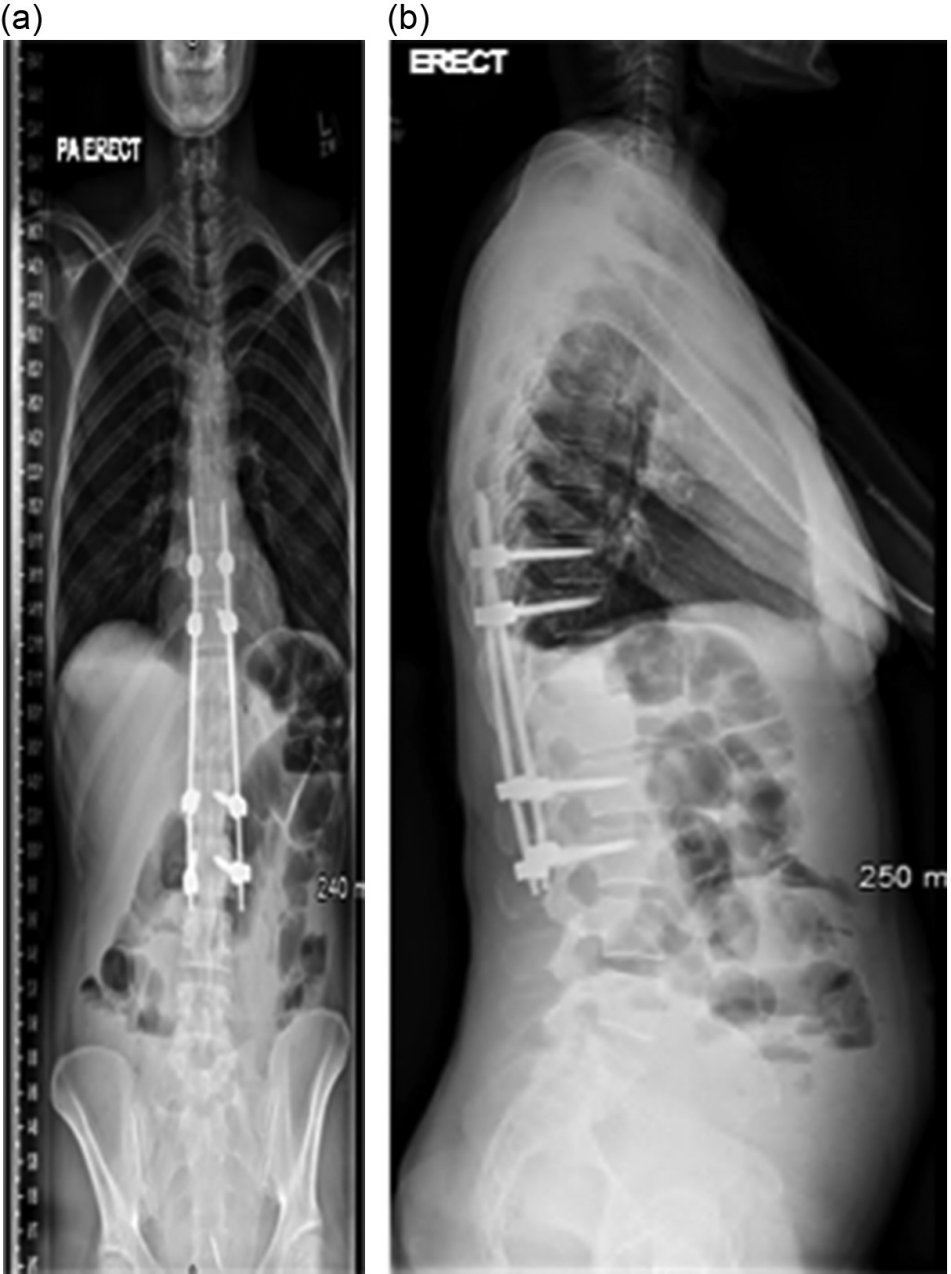
(a) Anteroposterior radiograph of the whole spine. (b) lateral radiograph of the whole spine. Stabilisation with screws and rods can clearly be seen from T10 to L3.

The patient underwent computed tomography (CT)-guided vertebral biopsy, with one biopsy and one pus sample being sent to the Microbiology Department at the Royal Free Hospital, London, UK. Six days post-biopsy, the patient underwent percutaneous posterior spinal stabilisation with bilateral pedicle screws and interconnecting rods from the tenth thoracic vertebra to the third lumbar vertebra (Fig. 2). Intraoperative samples were also received as the patient had not commenced antimicrobial therapy within this time. Samples included one fluid, one pus and one tissue sample. At the time of stabilisation surgery, there was no growth of any organisms, therefore, the patient was treated empirically post-operatively with intravenous teicoplanin (10 mg kg^−1^ 12 hourly for three doses and then 10 mg kg^−1^ once daily), piperacillin-tazobactam (4.5 g three times daily) and two doses of amikacin (15 mg kg^−1^ once daily), which is the standard antibiotic protocol for this centre.

The CT-guided pus sample received a Gram stain, which showed no organisms and few white blood cells. CT-guided and intraoperative samples were processed using glass Ballotini bead homogenisation with inoculation onto blood agar aerobically, chocolate agar in 5 % CO_2_ and fastidious horse blood agar anaerobically (in anaerobic jars), all at 35 °C, for 48 h incubation (cultures remained negative at 48 h). Homogenised samples were also inoculated into Bactec blood culture continuous monitoring liquid enrichment bottles for an extended fourteen day incubation based on the most current Public Health England Standards for Microbiology Investigation (SMI) for Investigation of Orthopaedic Implant Associated Infections [6]. Based on these SMI, only one enrichment method is required for routine diagnostic culture. The anaerobic Bactec bottles flagged positive (eleven days for the biopsy sample, three days for the intraoperative samples) and two metronidazole-sensitive anaerobic isolates were grown on fastidious horse blood agar (Oxoid, Basingstoke) from subculture. MALDI-ToF MS identified the two anaerobic isolates as *P. micra* and *Fusobacterium* species, and the isolates were sent to the Public Health England Anaerobic Reference Unit, Cardiff, UK, for formal identification and antimicrobial susceptibility testing. The reference unit identified the isolates as *F. nucleatum* and *P. micra*; both sensitive to amoxicillin, co-amoxiclav, clindamycin and ertapenem.

## Diagnosis

The patient’s diagnosis is polymicrobial spondylodiscitis of the tenth thoracic to the third lumbar vertebrae due to *P. micra* and *F. nucleatum*.

## Treatment

Upon identification of the isolates by MALDI-ToF MS, the patient commenced empirical intravenous ertapenem, 1 g once daily, delivered by a peripherally inserted central line catheter (PICC), and oral clindamycin, 450 mg four times daily, for a total of six weeks.

## Outcome and follow up

At her six week follow-up appointment in the bone and joint infection clinic, the patient reported no spinal pain, with a tender but non-erythematous operative site. Repeat X-rays showed no signs of further infection, and the patient’s CRP remained <10 mg l^−1^ and her white blood cell count was within normal reference limits. The patient was discharged from the bone and joint infection clinic as a treatment success.

## Discussion

Discitis is most frequently caused by aerobic bacteria such as *Mycobacterium* *tuberculosis*, *Staphylococcus aureus* and coagulase-negative staphylococci [7]. The anaerobes that most often cause anaerobic discitis are *Bacteroides* species and *Propionibacterium acnes* [7]. A review of the literature has shown that there have been very few cases [8–21] caused by *P. micra* or *F. nucleatum*, and only one case of discitis has been reported as polymicrobial with the isolation of both *P. micra* and *F. nucleatum* [8].

A slight majority of cases affected men (eleven out of eighteen [8–12, 14–17, 21]), and the mean age of patients was 62 years (range eight to eighty-five years, median age sixty-two years), which suggests that age and male sex may be contributing factors. The case described here had no known focus of infection, as the patient was in good health and had no known disease that might predispose her to transient bacteraemia from the mouth, gut or genital tract. The only predisposing factor that could have contributed to infection in this case was the injury to the back eight weeks prior to presenting at hospital or her intrauterine device. Supposition of source in this case is odontogenic or genital. The cases in the literature often (six out of eighteen cases [8, 10, 14, 19, 20]) cited an oral source for infection and periodontal disease, with a mean time from onset of symptoms to diagnosis of seven weeks. One of these cases [14] also cited a back injury prior to the onset of symptoms.

Seven of the reviewed cases [10, 12, 19, 21] utilised blood cultures for identification of causative organisms. The yield from blood cultures can range from 40–60 % in haematogenous pyogenic spondylodiscitis [22], and there have been reports of polymicrobial infections being missed when blood culture sampling is the only microbiological investigation [23]. In this case, the patient had a pre-operative blood culture taken to rule out bacteraemia, which remained negative at five days incubation. The yield of percutaneous biopsy is varied, with one group claiming a 75 % yield of positive results in 101 patients who all received biopsy [24]. For chronic discitis cases where the possibility of bacteraemia may be low, biopsy may provide greater results.

The under-diagnoses of anaerobes as a cause of spondylodiscitis may be due to the difficulty in growing these fastidious organisms. It is possible, had the solid media cultures been extended to five days incubation, that we may have isolated these anaerobes from solid agar culture; however, continuous monitoring blood culture systems have been shown to have a greater sensitivity than fastidious agar plates [6]. Effective empirical treatment of discitis requires a rapid identification to the species level. As molecular methods become more available in routine diagnostic laboratories, such as 16S rRNA PCR and mass spectrometry methods, such as MALDI-ToF MS, the reporting rates of difficult to culture anaerobes may increase. In the literature reviewed here, MALDI-ToF MS and 16S PCR were both utilised five times each [8, 9, 11, 14, 15, 17, 18] to provide identification. Antimicrobial treatment in our case was successfully guided by the fast turnaround of the MALDI-ToF MS result, as there was an unforeseen delay in obtaining the reference laboratory identification and sensitivities of the isolates. The empirical treatment chosen here was based on good anaerobic cover (ertapenem) with good coverage of any possible penicillin resistance, which has been reported in *P. micra* elsewhere [25], and good bone penetration (clindamycin).

In summary, this case has revealed that *P. micra* and *F. nucleatum*, organisms that are common oral commensals, can jointly cause spontaneous infection of the vertebrae, and that infection is rare but not uncommon in younger patients with no immunosuppression. Successful identification and subsequent treatment of these organisms relies on newer methods, such as anaerobic Bactec culture with extended incubation to fourteen days and MALDI-ToF MS.
